# A simple model of the right atrium of the human heart with the sinoatrial and atrioventricular nodes included

**DOI:** 10.1007/s10877-013-9429-6

**Published:** 2013-02-22

**Authors:** Piotr Podziemski, Jan J. Żebrowski

**Affiliations:** Faculty of Physics, Warsaw University of Technology, Koszykowa 75, 00-662 Warsaw, Poland

**Keywords:** Cardiac modelling, Right atrium, Cardiovascular physiology, Mathematical modelling, Atrial arrhythmia

## Abstract

Existing atrial models with detailed anatomical structure and multi-variable cardiac transmembrane current models are too complex to allow to combine an investigation of long time dycal properties of the heart rhythm with the ability to effectively simulate cardiac electrical activity during arrhythmia. Other ways of modeling need to be investigated. Moreover, many state-of-the-art models of the right atrium do not include an atrioventricular node (AVN) and only rarely—the sinoatrial node (SAN). A model of the heart tissue within the right atrium including the SAN and AVN nodes was developed. Looking for a minimal model, currently we are testing our approach on chosen well-known arrhythmias, which were until now obtained only using much more complicated models, or were only observed in a clinical setting. Ultimately, the goal is to obtain a model able to generate sequences of RR intervals specific for the arrhythmias involving the AV junction as well as for other phenomena occurring within the atrium. The model should be fast enough to allow the study of heart rate variability and arrhythmias at a time scale of thousands of heart beats in real-time. In the model of the right atrium proposed here, different kinds of cardiac tissues are described by sets of different equations, with most of them belonging to the class of Liénard nonlinear dynamical systems. We have developed a series of models of the right atrium with differing anatomical simplifications, in the form of a 2D mapping of the atrium or of an idealized cylindrical geometry, including only those anatomical details required to reproduce a given physiological phenomenon. The simulations allowed to reconstruct the phase relations between the sinus rhythm and the location and properties of a parasystolic source together with the effect of this source on the resultant heart rhythm. We model the action potential conduction time alternans through the atrioventricular AVN junction observed in cardiac tissue in electrophysiological studies during the ventricular-triggered atrial tachycardia. A simulation of the atrio-ventricular nodal reentry tachycardia was performed together with an entrainment procedure in which the arrhythmia circuit was located by measuring the post-pacing interval (PPI) at simulated mapping catheters. The generation and interpretation of RR times series is the ultimate goal of our research. However, to reach that goal we need first to (1) somehow verify the validity of the model of the atrium with the nodes included and (2) include in the model the effect of the sympathetic and vagal ANS. The current paper serves as a partial solution of the 1). In particular we show, that measuring the PPI–TCL entrainment response in proximal (possibly-the slow-conducting pathway), the distal and at a mid-distance from CS could help in rapid distinction of AVNRT from other atrial tachycardias. Our simulations support the hypothesis that the alternans of the conduction time between the atria and the ventricles in the AV orthodromic reciprocating tachycardia can occur within a single pathway. In the atrial parasystole simulation, we found a mathematical condition which allows for a rough estimation of the location of the parasystolic source within the atrium, both for simplified (planar) and the cylindrical geometry of the atrium. The planar and the cylindrical geometry yielded practically the same results of simulations.

## Background

Disorder of the pump action of the heart is often caused by abnormal electrical activity within the heart atria. By modeling this activity, we can study the physical processes responsible for the properties of the dynamics of the heartbeat and look for the sources of the disorders of heart rhythm.

For a computational modeling of the atrial arrhythmias attention needs to be focused on the anatomy and electrophysiology of the atrium. In various studies, the geometry of the modeled anatomy varies from pseudo one dimensional models [19] through cylindrical-like structures [7] to detailed three-dimensional models including the atrial wall and fiber direction [30]. A recent article of Dössel et al. [11] shows the current state-of-the-art in the computational models of atria and presents a list of the 3D geometrical atrial models developed recently. The influence of the atrial anatomy on the arrhythmias from the clinical electrophysiological point of view have been summarized recently in the review article by Garcia-Cosio et al. [16]. The latest computational approaches to guiding arrhythmia therapy are presented in a recent review by Roberts et al. [27]. As for the electrophysiological models of the atrial tissue, they are still being studied thoroughly since the first model of human atrial tissue presented in [9]. Recent studies presenting the role of Ca^2+^ transport in normal and remodeled human atrium by Grandi et al. [17] and the role of the Na^+^K^+^ pump current in determining human atrial electrophysiology by Sánchez et al. [29] indicate, that action potential duration (APD) restitution properties for the normal and a remodeled tissue show strong differences from patient to patient. Moreover, the computational models of human atrial electrophysiology show different APD and conduction velocity (CV) restitution properties [11]. Thus, simpler models, which allow to adjust easily restitution properties of the tissue, are needed to effectively simulate cardiac tissue. An example of such a model is the 3V model developed by Fenton and Karma [12]. For the pacemaker tissue, on the other hand, currently only few models of human sinoatrial nodal (SAN) tissue exist, with two reported in [11]. Also, models of human atrioventricular nodal (AVN) tissue are still to be developed (e.g. Inada et al. [19] developed a model of the rabbit AVN).

Computational models should become a part of an integrative approach for the validation of cardiac electrophysiology covering simulations, experiments and clinical studies [4]. However, most of the models mentioned in [11] are highly computationally complex and cannot be used to simulate long time dynamical properties of the heart (several hundreds to thousands of heartbeats). Moreover, the introduction of the effect of the autonomic nervous system into those models is difficult and rarely studied, while the focus of the emerging new paradigm is the autonomic nervous system and its important role in various pathological states [24, 25, 31]. A recent study by Muñoz et al. [24] indicates that the onset of atrial arrhythmias may be evoked by the autonomic modulation of the rabbit sinoatrial node activity. The role of vagal stimulation in promoting the induction of AF was studied in a simple model of the vicinity of the myocardial sleeves of the pulmonary veins by Zemlin et al. [37]. Such an approach to simulation, in which simplifications are justified by the hypothesis to be tested, may be the key to connect the computational approach with the newest clinical and medical results.

## Introduction to the study

In our previous studies, we developed a nonlinear oscillator model for the sinoatrial and atrioventricular tissue. The model reproduced pacemaker action potential sequences with the proper firing frequency and action potential shape. It reproduced the way the vagal activity modulates the heart rate and several phenomena well known in cardiology, such as the vagal paradox [35], or in an extended setting-concealed conduction effects in the atrium [36]. These results demonstrated that such simple, phenomenological nonlinear models of the heart conduction system can be a source of insight and a tool to distinguish, describe, and understand cardiovascular phenomena in spite of the simplifications used and the lack of ion channel properties. Here, we describe simulations of the electrical activity of the human right atrium with the previously developed models of sinoatrial and atrioventricular tissue included.

There are three main motivations for the present study:

1. Only a few models of the human SAN tissue and no model of human AVN have been published [11, 19]. Still, there are many supraventricular tachycardias (SVT) in which the nodal tissue plays a major role, such as the AV nodal reentrant tachycardia, AV reentrant tachycardia or AV conduction blocks. Typical consequences of those arrhythmias are a decline in the quality of life, tachycardia-induced cardiomyopathy or bradycardias requiring pacemaker implantation [22]. A model of the atria that would include SAN and AVN would have the ability to generate sequences of RR intervals during arrhythmia, which is relevant for the interpretation of ECG monitoring signals. Studies of the effect of the autonomic nervous system on atrial electrophysiology and of its important role in various pathological states also would require the incorporation of the nodal tissue into the models of atrial activity. Such combined models containing the atrium as well as the nodes need to be developed.
2. Human heart rate variability is one of the major factors in the effective functioning of the cardiovascular system, both in the normal and in the arrhythmic state. Usually, the existing physiological models including cell membrane ion channels are too complex to allow to combine an investigation of long time dynamical properties of the heart rhythm with the ability to effectively simulate cardiac electrical activity during arrhythmia. As a consequence, very rarely are such models used to obtain heart rate variability comparable with portable ECG recordings. Note that the physiological (or sinus) heart rhythm is mostly defined by the dynamics which occurs within the right atrium.
3. The mechanism of an arrhythmia can be identified in a clinical electrophysiology (EP) lab by entrainment [1, 10, 32]. Methods or entrainment protocols that allow to recognize and locate the source of the arrhythmia and which also help to locate the mapping catheter in relation to the arrhythmia circuit are still being developed. One way to do this is to use computer simulations of the electrical activity of the human heart. Models exist that are designed for this purpose [3, 5]. However, models with a simplified anatomy may allow to test and visualize new and existing entrainment protocols and present spatiotemporal relations between pacing and tachycardia within a reasonable computer time.

To address those problems we describe a model of the right atrium combined with a generic model of the nodal tissue, specifically designed to qualitatively reproduce mesoscopic characteristics of pacemaker cell dynamics, including action potential duration (APD) restitution curves and phase response characteristics. The parameter space of the model is small (7 parameters) to easily adjust the APD and CV restitution properties or frequency. As the effect of atrial muscle tissue properties on the arrhythmia (i.e. during AF remodeling) is well studied, here we focus on the study of phenomena due to the properties of nodal tissue rather than atrial working muscle tissue. Because of that, we use simple models for the atrial muscle tissue: the FitzHugh–Nagumo [13] nonlinear model belonging to the Liénard class of equations and, independently, the Fenton and Karma [12] ion-channel model of atrial tissue.

We test our model on 3 hypothesis concerning electrical conduction phenomena in the RA:

1. A regular parasystolic source in the right atrium, which has a period larger than that of the sinus rhythm, may alter the properties of heart rhythm in a characteristic nonlinear way depending on its location.
2. Alternans in the conduction through the AV junction during atrio-ventricular orthodromic reciprocating tachycardia (ORT) is a function of the restitution properties of the AV node and does not require a dual pathway to the AVN.
3. During atrioventricular nodal reentrant tachycardia (AVNRT), entrainment from the RA may suppress the arrhythmia depending on the period of entrainment. Moreover, entrainment pacing from the RA may provide a support pacing maneuver for the common ventricular overdrive pacing method (as described in [18]) which is used to distinguish AVNRT from other atrial tachycardias. In some cases ventricular overdrive pacing does not provide a clear discrimination [34]. A similar method, in which pacing within the RA helped to distinguish RA tachycardia from LA tachycardia, was published by Miyazaki et al. [23].

The model in its present state of development may be used to demonstrate conduction phenomena in the atrium. With a more realistic geometry and description of the atrium tissue properties, the model will be developed as a support tool for procedures used in an electrophysiology lab.

### Organization of the paper

The mathematical foundations of the model are explored and shown in Sect. 3. Atrioventricular nodal tachycardia, ventricular-triggered atrial pacing and atrial parasystole are investigated in Sect. 4. In Sect. 5: Conclusions, the results are summarized and their applicability is discussed.

## Methods

### Model of the right atrium of the heart

One of the main motivations for this study was to produce a computational model of the RA, which in the future will allow an investigation of long time dynamical properties of heart rhythm. Consequently, certain simplifications and assumptions had to be made.

#### Justification of simplifications:

1. The human atrium wall has a thickness between 1 and 3 mm [11], so in most of the cases the pathological phenomena will occur on the surface formed by atrial tissue and can be analyzed on a two dimensional surface.
2. The minimal plausible geometry for the atrium tissue is difficult to define. Here, we compare the results obtained in two geometries: an idealized cylindrical surface and a two dimensional, planar geometry of the atrial tissue.
3. As research in the field of human nodal tissue (both SAN and AVN) is not abundant [11], and its APD and CV restitution properties vary significantly between patients with heart disorders, a generic, qualitative model with a low number of parameters for both nodes is used. The restitution properties, APD length and period of firing can be easily adjusted in this model. The model can be coupled with autonomic regulation models (coupling between nodal tissue and vagal stimulation was tested in [35],where the physiological phenomenon of vagal paradox was reproduced).
4. The ion channel modeling of muscle tissue requires a relatively large number of parameters. These parameters allow to change the properties of the simulated tissue depending on the activity of the different ion species. Thus, they are usefull for e.g. pharmaceutical research. However, to be used for clinical purposes, the values of these parameters should be adjusted as action potential duration (APD) restitution properties for the normal and a remodeled tissue show strong differences from patient to patient and from model to model [17, 29]. Given the large number of parameters in ion channel models this is not an easy task. Thus, simpler models, which allow to adjust easily restitution properties of the tissue, are needed to effectively simulate cardiac tissue. For the atrial tissue two simple models were chosen: the FitzHugh–Nagumo model [13] and the Fenton and Karma [12] ion-channel minimal model of cardiac tissue. The former is an oversimplified model but has the advantage that its equations belong to the same Liénard class as the model for nodal tissue used here. This allows to have a single class of equations covering all the types of tissue in the model (working muscle and nodal). The latter model has proper restitution properties and a still low parameter space. The results for both models are compared for each case of simulation presented in this paper. Note, that most of the phenomena analyzed in this paper do not occur on the time scale where specific APD and CV restitution properties of the atrial muscle tissue can lead to alternans or affect the simulated phenomena in qualitative way.
5. We assume that any pathology studied here appears only in the right atrium, where the SAN and AVN nodes are located, while other parts of heart (left atrium and ventricles) work properly. Thus, we can assume that the intervals between consecutive action potentials (interspike intervals, ISI) exiting the AV node are comparable to RR intervals measured in an ECG recording.
6. Because of the requirement for fast calculations, a monodomain model of the conduction and the Euler explicit integration scheme were used [11].

#### Geometry of the model 

**Fig. 1:**
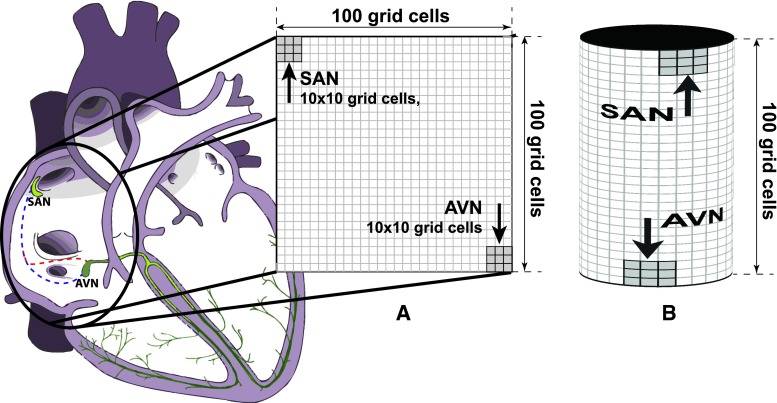
The model setting. We define a simplified model of the atria, consisting of 100 × 100 simulation cells divided into dedicated regions: the sinoatrial (*SAN*), the atrio-ventricular (*AVN*) nodes and regions of normal atrial conductive tissue in between. The implementation of the model allows to put non-conductive regions inside the model matrix to simulate anatomical details necessary for some arrhythmias to occur. These are e.g. coronary sinus, tricusipd valve, the fast and slow pathway for AVNRT. We assume that any pathology occurs only in the right atrium , while the left atrium and the ventricles work properly. Thus, we can assume that the intervals between consecutive action potentials exiting the AV node are equivalent to the RR intervals measured in the ECG. Two tissue geometries were used in the study: a two dimensional plane (**a**) and cylindrical surface (**b**). For all of the simulations a computational cell size of $\Updelta x =0.08$  cm was used, forming a domain of 8 cm × 8 cm. For the cylindrical geometry, this resulted in the cylinder diameter equal to 2.6 cm. For a discussion of the dimensions, see Sect. 3.1

Two tissue geometries were used in the study: a two dimensional plane and a cylindrical surface (Fig. 1). The surface of both was composed of a matrix of 100 × 100 computational cells with regions devoted to the sinoatrial node (SAN), the atrio-ventricular node (AVN), regions of normal atrial conductive tissue and, in some cases, non-conductive regions (such as the coronary sinus). Other anatomical details may be easily introduced in the model if necessary. Each computational grid cell represents a group of tissue cells and is modelled by a set of ordinary differential equations. For all of the simulations, a computational cell size of $\Updelta x =0.08$  cm was used, forming a domain of 8 cm × 8 cm. For the cylindrical geometry, the resultant diameter of the cylinder was 2.6 cm. This is consistent with the lower reference value for the RA minor dimension [28]. The resultant 8 cm cylinder height is more than the reference value for the RA major dimension (5.3 cm in the study by Rudski et al. [28]), however both cylinder diameter and height were chosen so as to compensate for the curvature of the inner surface of the atrium. For a schematic of the model setting, see Fig. 1.

#### Conduction properties

All grid cells are coupled diffusively at the interfaces (monodomain conduction model [11]). The parameters responsible for the conduction (see below) were chosen to obtain the velocity of the activation wave equal to 60 cm/s at a basic cycle length of 840 ms which is slightly lower than that shown by Dössel et al. in [11]. The CV restitution curve for nodal tissue model was measured in a tissue strip of 8 cm × 0.32 cm. To clearly present the transition from sinus rhythm to AVNRT, a lower velocity of the activation wave (35 cm/s) was chosen for the FitzHugh–Nagumo model for the simulation of AVNRT. Though low, such a magnitude of velocity is comparable with the velocities obtained in pathological states when atrial remodeling occurs [11] (for patients without AF, the conduction velocity in the atrium is between 51 and 120 cm/s while under pathological conditions—between 37 and 133 cm/s [11]). Von Neumann (zero flux) boundary conditions were chosen at the border of any non-conducting region included in the computation and at the edges of the model.

#### Time-scale properties

For the Fenton–Karma model, the APD duration was between 180 and 230 ms for different pacing cycles. For the SAN and AVN tissue model coupled with the Fenton–Karma atrial tissue model, the APD duration between 200 and 250 ms was obtained for different pacing cycles (the parameters are discussed below). For the FitzHugh–Nagumo model it was impossible to obtain such values (the FitzHugh–Nagumo model does not reproduce properly the action potential duration), so a smaller APD was used—in the range 70–120 ms. As a result, for the SAN and AVN tissue model coupled with the FitzHugh–Nagumo atrial tissue model, we had to use a shorter APD duration between 80 and 140 ms (the parameters are discussed below, the APD/DI and CV restitution curves for nodal tissue are presented in Fig. 8). For the SAN nodal model coupled with the tissue, a sinus cycle of 800–840 ms was set, for the AVN a cycle of 1,300 ms was set.

#### Mathematical model of the SAN and AVN pacemaker tissue: van der Pol-Duffing equation (Liénard class of ODE)

Equations of different forms but all belonging to the single class of Liénard equations have been used to model biological, chemical or physical processes, varying from microtubule kinetics to neuron firing [14]. Moreover, many complicated models can be effectively simplified to the form of the Liénard equation e.g. the Morris-Lecar ionic channel model for neurons, a model of the Bielusov-Zhabotynski reaction kinetics and other models [14]. The model of the nodal tissue here is based on a neuron model by Postnov et al. [26]. The model is, in fact, a van der Pol-Duffing equation which also belongs to the class of Liénard equations. Note that, although the model is dimensionless, the variable *v*—the magnitude of the action potential—may be easily rescaled to the physiological range by a linear scaling function. With some modifications, it was used to model the conduction system of the heart in [35]. The action potential obtained from the model exhibits the main properties of the action potential of the sinoatrial or atrio-ventricular nodal tissue measured in animal experiments: the shape, the refractory time and the diastolic time.

The model of [35] was not able to reproduce fully the physiological response of the sinoatrial nodal tissue to an external stimulation. The most important limitation was that it was able to reproduce only one type of phase response of the two types which occur in nature for the AV node. Taking into account the the general mathematical properties of the Liénard equations and of the van der Pol-Duffing equation, we applied the Liénard transform [21] to remedy this flaw. Application of this transform allows to retain all the intrinsic properties of the model and obtain its response to external stimulation similar to natural. The final form of the van der Pol-Duffing model after the Liénard transformation is given by the Eq. (1):

1 $$ \left\{ {\begin{array}{*{20}l}    {\frac{{dv}}{{dt}} = \frac{\alpha }{\mu }\left( {v - \left( {\frac{1}{3}v^{3} } \right) - w} \right) + \nabla \left( {D\nabla v} \right),} \hfill  \\    {\frac{{dw}}{{dt}} = \frac{f}{\alpha }v(v + d)(v + e)}. \hfill  \\   \end{array} } \right. $$

where *v* = *v*(*x*, *y*) is the activation variable corresponding to the action potential of the tissue element located at (x, y), *w* = *w*(*x*, *y*) is the control variable in the tissue element at (x, y), α is the damping constant, μ determines the amplitude of oscillations while the parameter *f* enables to rescale the frequency without changing the structure of the phase space of the system. The system has three fixed points residing on the horizontal axis *v*: an unstable focus at *v* = 0 , a saddle at *v* = −*d* and a stable node at *v* = −*e*, where *d* and *e* are parameters. Note that the diffusive coupling term $\nabla\left(D\nabla v\right)$ with *D* = *D*(*x*, *y*) was inserted into the model and that the differential equation for *v* matches the corresponding equation in the FitzHugh–Nagumo model. After discretization to a two dimensional computational grid, the van der Pol-Duffing equations become:

2 $$ \left\{\begin{array}{l} \frac{dv_{i,j}}{dt} \, = \, \frac{\alpha}{\mu}\left(v_{i,j} - \frac{1}{3}v_{i,j}^{3}- w_{i,j}\right)+ D_{i,j}\left(v_{i+1,j}- v_{i,j}\right) \\  \quad \quad- D_{i-1,j}\left(v_{i,j}- v_{i-1,j}\right) + D_{i,j}\left(v_{i,j+1}- v_{i,j}\right) - D_{i,j-1}\left(v_{i,j}- v_{i,j-1}\right),\\ \frac{dw_{i,j}}{dt} \, = \,  \frac{f}{\alpha}v_{i,j}(v_{i,j}+d)(v_{i,j}+e).\\ \end{array}\right. $$

where *i*, *j* = 1,…, *n* are indexes of a grid element.

#### Atrial muscle tissue: FitzHugh–Nagumo equation (Liénard class of ODE)

In order to be able to study the model using a single class of equations covering all the types of tissue in the model (working muscle and nodal), we chose the simple FitzHugh–Nagumo model for the atrial tissue. To verify whether its use is reasonable for the simulated phenomena, we compare these simulations with the results for the Fenton–Karma 3V ionic model [12] of cardiac tissue—in the next section. The FHN model captures the key features of excitable media and is widely used as a simple model of cardiac muscle electrical activity. The equations of the discretized FHN model are:

3 $$ \left\{\begin{array}{l} \frac{dv}{dt} = \alpha \left(v - \frac{1}{3}v^{3}- w\right) + \nabla\left(D\nabla v\right) ,\\ \frac{dw}{dt} = \mu(v+\beta-\gamma w).\\ \end{array}\right. $$

where *v* = *v*(*x*, *y*) is the activation variable basically corresponding to the action potential of the element (x, y), *w* = *w*(*x*, *y*) is the slow control variable at that element. After discretization to a computational grid, the FitzHugh–Nagumo (FHN) equations become:

4 $$ \left\{\begin{array}{l} \frac{dv_{i,j}}{dt} = \alpha\left(v_{i,j} - \frac{1}{3}v_{i,j}^{3}- w_{i,j}\right) + D_{i,j}\left(v_{i+1,j}- v_{i,j}\right) - D_{i-1,j}\left(v_{i,j}- v_{i-1,j}\right)\\ \quad \quad+ D_{i,j}\left(v_{i,j+1}- v_{i,j}\right) -D_{i,j-1}\left(v_{i,j}- v_{i,j-1}\right) ,\\ \frac{dw_{i,j}}{dt} =\mu\left(v_{i,j}+\beta-\gamma w_{i,j}\right).\\ \end{array}\right. $$

where *i*, *j* = 1, …, *n* are indexes of a grid element. An external coupling in the form of the diffusive terms was added and does not occur in the original Liénard equations.

#### Atrial muscle tissue: Fenton—Karma 3V model

The Fenton–Karma 3V model, first reported in [12], is a three variable ion-channel model of cardiac cell electrical activity. It was designed to reproduce the APD and CV restitution curves rather than the shape of action potential, for the restitution properties indicate the dynamics of the depolarization wavefront and conduction—fundamental quantities for the modeling of wave dynamics. The equations and different parameter sets, along with the APD and CV restitution curves corresponding to those parameters can be found in [12]. Parameters for the restitution curves of the Luo-Rudy-I model [12] were used here with minor modifications to model the atrial muscle tissue action potential.

#### Model parameters

Two versions of the RA tissue model were used consistently throughout the study: one with with FitzHugh–Nagumo model of tissue and the second with the Fenton–Karma model of tissue. Parameters for the former are presented in Table 1, and for the latter in Table 2. The membrane potential *v* was kept dimensionless for all models. Choosing the parameters properly, the intrinsic dimensionless time of the pacemaker models and of FitzHugh–Nagumo model was set to be numerically comparable with the time scale usually encountered in reality (in ms), so no time scaling is needed. For the Fenton–Karma model, the time is in units of ms.

**Table 1:** Parameters for the RA model with the Fenton–Karma model of atrial tissue

Parameter	Value	Parameter	Value	Parameter	Value
*Fenton–Karma 3V model*					
*g*_*fi*_	4.4	τ_*r*_	130	τ_*si*_	127
τ_0_	12.5	τ_*v*_^+^	10	τ_*v*1_^−^	18.2
τ_*v*2_^−^	18.2	τ_*w*_^+^	1020	τ_*w*_^−^	80
*u*_*c*_	0.13	*u*_*v*_	–*	*u*_*c*_^*si*^	0.85
*SAN and AVN pacemaker model*					
α	0.1	μ	0.2	*f*	0.0002
*d*	3	*e* for SAN	2	*e* for AVN	1.5

The time is in $ms,\,C_{m} = 51\,mF/cm^{2},\,\tau_{d}=C_{m}/g_{fi}$ with *g*_*fi*_ in mmho/cm^2^, and *k* = 10. Time is dimensionless for the pacemaker model but its scale is numerically comparable to that obtained in reality (in ms). The diffusive coupling constant D was set to $0.25\,cm^{2}/s,$ if not stated otherwise; the constant D was set so as to obtain the conduction velocity (CV) of $60\,cm/s$ at a basic cycle length of 840 ms. To compensate for the difference in diastolic potential at the border between nodal and atrial tissue for both models, a linear rescaling *v* = (0.5*v* + 0.5) was performed. The membrane potential *v* was kept dimensionless as in [12] and [35]

* The value of *u*_*v*_ for the chosen parameters does not need to be defined [12]

**Table 2:** Parameters for the RA model with the FitzHugh-Nagumo model of atrial tissue

Parameter	Value	Parameter	Value	Parameter	Value
FitzHugh–Nagumo model					
β	0.4	γ	1.3	α	0.3
μ	0.02				
SAN and AVN pacemaker model					
α	1.0	μ	1.0	*f*	0.0003
*d*	3	*e* for SAN	5.3	*e* for AVN	3.5

Time is in dimensionless units for both FitzHugh–Nagumo model and pacemaker model but the time scale is numerically comparable to that obtained in reality (in ms). The diffusive coupling constant D was set to 0.5 cm^2^/s if not stated otherwise; the membrane potential *v* was kept dimensionless as in [12] and [35]

#### Implementation of the model

**Fig. 2:**
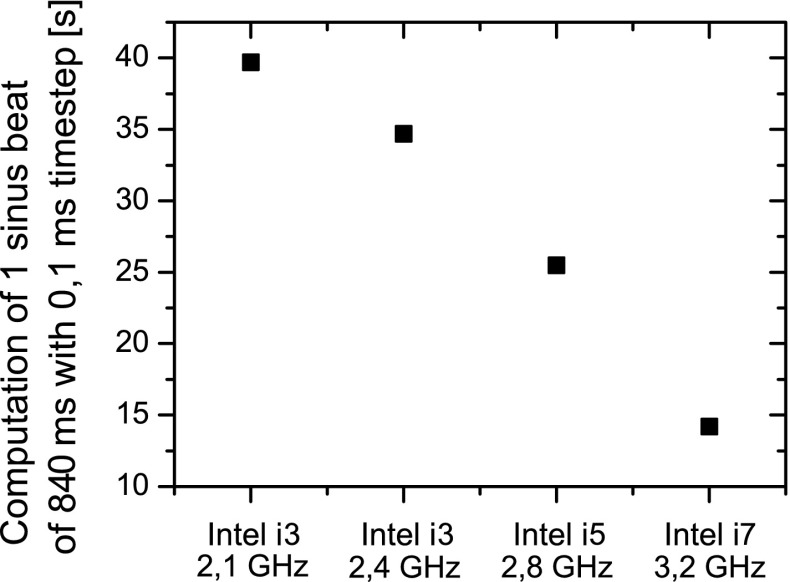
Comparison of computational time of a single cycle of sinus rhythm using other PC processors for $\Updelta t=0.001$ s

The model was implemented in C++ using the Qt Graphical User Interface. The Euler explicit integration scheme was used throughout this paper with a time step $\Updelta t=0.001$ s. In the initial stage of model development 4th order Runge-Kutta fixed step integration was used but it was quickly found that the Euler scheme gives equivalent results and it is somewhat faster. The model runs as a standalone application on a single core of a 4-core Intel i7 PC 3.2 GHz processor of a desktop PC and was provided with a graphical interface allowing to define the anatomical details of the simulation and its parameters. A comparison of the computational time on other types of processors is provided in Fig. 2. The total time needed to conduct a single revolution of sinus rhythm was less than 15 s for $\Updelta t=0.001$ s, 7 s for $\Updelta t=0.002$ s, and 4 s for $\Updelta t=0.003$ s. This is at least an order of magnitude better than one cycle of sinus rhythm obtained in [24].

Higher time steps were not used to stay in the convergence region of the integration algorithm. Such a setting was chosen to optimize the programme to be run on a standard PC as a support or demonstration tool.

## Results and discussion

Looking for a minimal model, currently we are testing our approach on certain chosen well-known arrhythmias, which were obtained until now only using much more complicated models. To evaluate the model, three arrhythmia case studies were chosen for simulation: atrio-ventricular nodal reentrant tachycardia (AVNRT), AV orthodromic reciprocating tachycardia and atrial parasystole.

### AV nodal reentrant tachycardia

Atrio-ventricular nodal reentrant tachycardia (AVNRT) is one of the most common supraventricular arrhythmias that occur in the right atrium: a reentry loop is formed in the vicinity of the atrioventricular node, within the right atrium [18]. The reentrant loop involves two (or sometimes more) anatomical conduction pathways: the fast and the slow pathway. The fast pathway is usually located superior to the AV node. The slow pathway is located on the anterior site of the coronary sinus inferior to the AV node. A simulation of this arrhythmia was published by Inada et al. [19] but the model was limited to a 1-dimensional strip of rabbit tissue. Here, AVNRT is studied in the two geometrical settings of RA—the two dimensional tissue strip using the FitzHugh–Nagumo model of the atrium tissue and the cylindrical idealized geometry of the RA using the Fenton–Karma model of the tissue. In the first model, a transition between sinus rhythm and AVNRT is shown, in the second model we simulate entrainment pacing from the RA as a method to distinguish between AVNRT and other atrial tachycardia.

#### Two dimensional geometry in the FitzHugh–Nagumo model of tissue for the study of the induction of AVNRT

For the two dimensional geometry, the AVN and the SAN node were placed at the ends of the diagonal of the square tissue strip and separated by the coronary sinus with the diffusion coefficients chosen so as to simulate the difference between the pathways. The two-dimensional model allows insight into the dynamics of the action potential wave and the phase relations inside the atrium including the effect of anatomical details. To obtain the AV nodal reentry in a model, a few different methods may be used. One example is the double stimulation S1–S2 protocol [19] which simulates the effect of an ectopic beat. All the methods are functionally equal to a functional block in one of the pathways. For an AVNRT, in which the fast pathway conducts anterogradely while the slow pathway is the retrograde path, the following procedure was used:

1. At first, an action potential fails to enter the slow pathway due to a short time prolongation of the refractory period in the slow path. In the model, the effect was obtained by setting temporarily the parameter α of the FitzHugh–Nagumo model to *α* = 0.15 inside the whole nodal area, which effectively puts the model into the refractory state (by damping the incoming potential waves). In such a case, the action potential that propagates from the SAN will propagate only through the fast path to reach the AV node.
2. From the AV node a backward action potential will travel along the slow pathway, while the rest of the tissue is restored to the initial state (*α* = 0.3). By the time the action potential reaches the end of the slow pathway, the fast pathway becomes reexcitable—as does the rest of the atrium.
3. As a result, a backward conduction occurs towards the SAN and—more importantly—another propagation along the fast pathway also occurs. From that moment, AVNRT is stable

#### Simulation results

**Fig. 3:**
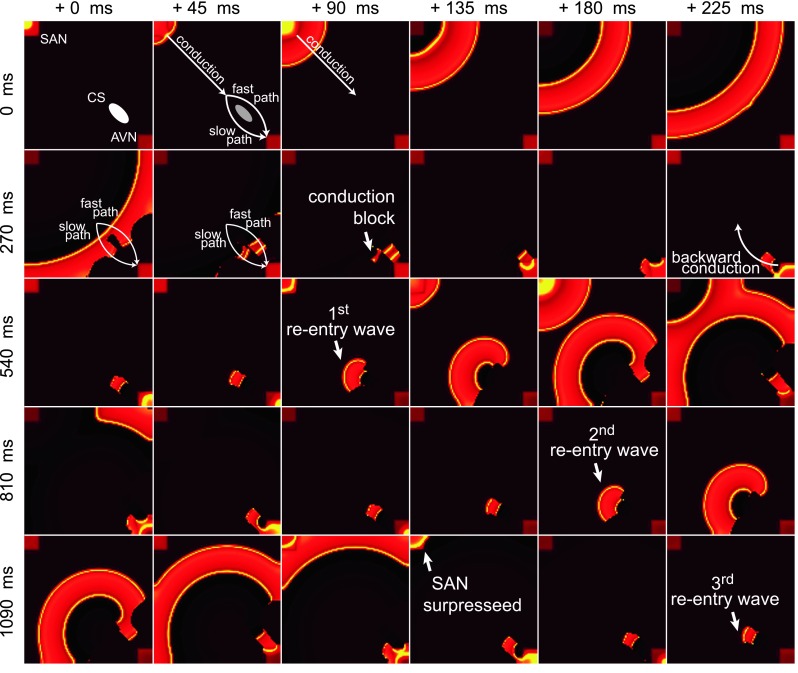
Simulation of atrio-ventricular nodal tachycardia. To simulate the AV node reentry, we prepared a dedicated model of the right atrium. In that model, the two anatomical pathways to the atrio-ventricular node region were introduced: a slow and a fast pathway, located at both sides of the non-conducting region containing the coronary sinus. The action potential propagating during the initiation and first moments of AVNRT can be seen. The* rows* are* labeled* by the simulation time at the* leftmost column* while the columns are marked with the time shift (45 ms) between them. At the beginning, the sinoatrial node produces an action potential, which travels through the atria towards the atrio-ventricular node (*t* = 45 ms:225 ms). Then, the action potential reaches the fast and the slow conducting pathways and enters them (*t* = 270 ms:360 ms). The refraction time within the inside the slow conduction pathway is briefly reduced resulting in a local functional block. Thus, the action potential propagates forward only through the fast (anterograde in this case) pathway (*t* = 360 ms:450 ms). Next, because of the excitation of the AV node, a backward (retrograde) conduction appears in the slow pathway (*t* = 495 ms:630 ms). The action potential travels backward through the right atrium and excites it while the loop of AVNRT is formed—the action potential repeatedly enters the slow anterograde pathway towards the AV node (*t* = 720 ms). 3 revolutions of AVNRT are shown in the figure. Note that after the second revolution, the activity of the sinoatrial node is completely suppressed by the reentrant wave

Results of simulation of the atrio-ventricular nodal tachycardia are presented in Fig. 3. The rows are labeled by the simulation time at the leftmost column while the columns are marked with the time shift (45 ms increments) between them. To obtain the type II AVNRT (“fast-slow”) in our model, it was enough to disrupt the conduction in the slow pathway. We did not need to use the double stimulation S1–S2 scenario as in [19]. The length of the time this pathway remained disrupted was not important. The phase at which it regained its normal state was crucial. We were able to find such a combination of the refraction periods and diffusion coefficients for which the reentry continued indefinitely. The parameters were set according to the Table 2. The diffusion coefficient in the whole atrium was set to 0.25 cm^2^/s and in the slow pathway area of 1.5 cm^2^ below the coronary sinus (CS) to* D* = 0.08 cm^2^/s). It is possible that type II AVNRT will always be more stable as the return path is slower, which makes the phenomenon less sensitive to the refraction time at the entry to the fast path–slow path junction.

#### AVNRT in an idealized cylindrical geometry using the Fenton–Karma 3V model of tissue

**Fig. 4:**
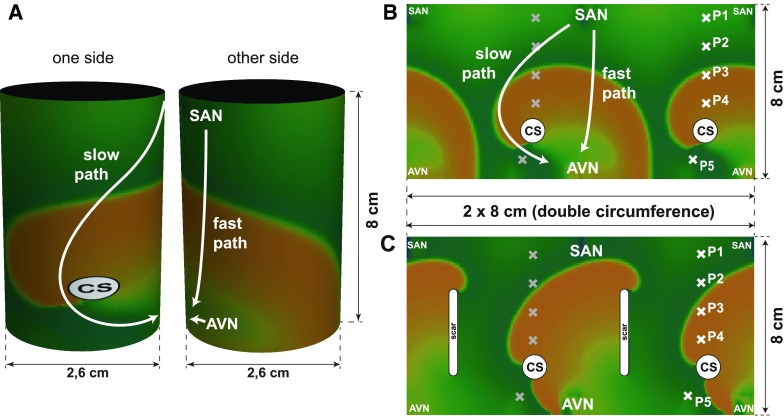
The cylindrical idealized geometry of RA (**a**). The AVN and the SAN were placed near the top and bottom edges of the model. The coronary sinus was set at a distance of 1 cm along the fast conduction pathway. The diffusion coefficients chosen so as to simulate the difference of the conduction in pathways (in the whole atrium *D* = 0.25 cm^2^/s, in the slow pathway area of 1.5 cm^2^ below the coronary sinus (*CS*) the conduction coefficient was three times smaller).** b**,** c** the projection of the surface of the cylindrical model for two cases studied to show the periodicity of cylindrical topology. Simulated entrainment electrode placement was marked by the* white crosses* *x* (*gray crosses* represent the same electrode positions as the white ones).** b** depicts the geometrical setting for the simulation of AVNRT while,** c** the geometrical setting for atypical RA flutter around scar tissue [8]).** b**,** c** The surface of the cylinder is unfolded twice. The double circumference projection was chosen to better depict the effect of periodic boundary conditions i.e. the cylindrical geometry. As consequence the images of CS, the scar and of the electrodes are given twice

Here, a comparison between PPI–TCL entrainment pacing response from the RA in an AVNRT arrhythmia and in an atypical RA flutter [8], around scar tissue is presented in a cylindrical geometry. The AVN and the SAN were placed near the top and bottom edges of the model as presented in Fig. 4. The coronary sinus was set 1 cm away along the fast conduction pathway. The diffusion coefficients was chosen so as to simulate the difference of the conduction in pathways (in the whole atrium 0.25 cm^2^/s in the slow pathway area of 1.5 cm^2^ below the coronary sinus (CS) the conduction coefficient was three times smaller). Entrainment electrodes placement was marked by the crosses *x* in Fig. 4. Figure 4b depicts a projection of the geometrical setting for the simulation of AVNRT, while Fig. 4c shows the projection for the atypical RA flutter around scar tissue.

#### Simulation results

**Fig. 5:**
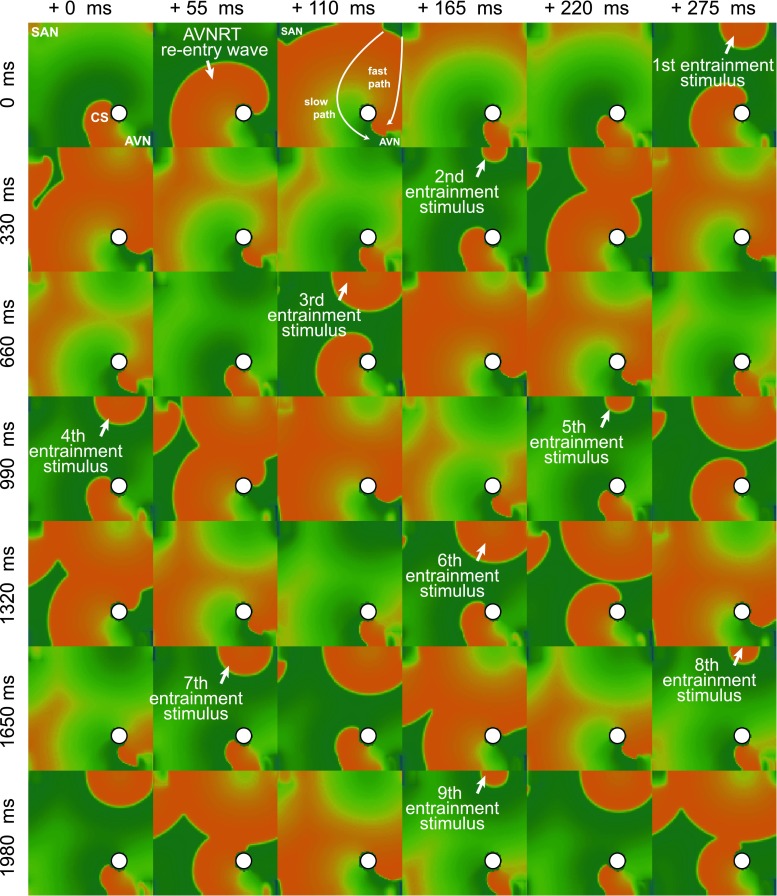
Simulation of atrio-ventricular nodal tachycardia entrainment. To simulate the AV node reentry, we prepared a dedicated model of the right atrium using a cylindrical idealized geometry shown in Fig. 4 where two anatomical pathways to the atrio-ventricular node region were introduced: a slow and a fast pathway, located at both sides of the non-conducting region containing the coronary sinus. The* rows* are* labeled* by the simulation time at the* leftmost column* while the columns are marked with the time shift (55 ms) between them. For the first 220 ms, one AVNRT revolution can be seen. Note the complex activation of the AVN tissue (*right bottom* corner of the pictures in the time strip). The AVNRT loop is qualitatively similar to the one achieved in the 2D plane geometry and the FitzHugh–Nagumo tissue model. At (*t* = 220 ms), entrainment pacing is initiated from the electrode placement marked as *P*1 in Fig. 4. After 9 pacing cycles, the AVNRT becomes fully entrained-the activation wave from the entrainment pacing site accelerates the conduction along AVNRT circuit

**Fig. 6:**
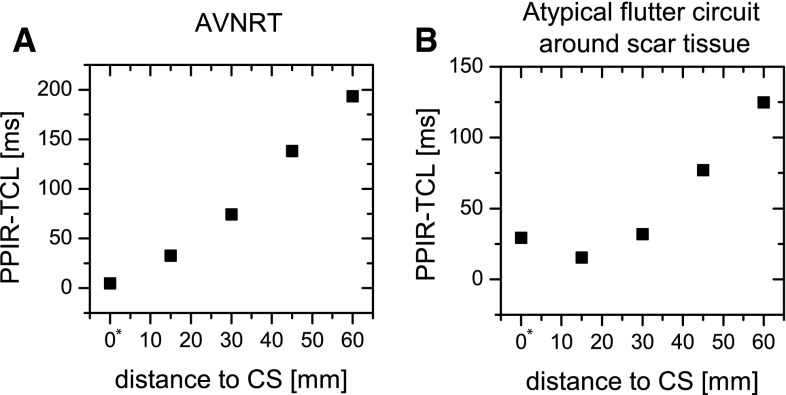
PPI–TCL intervals from the simulation of entrainment pacing of AVNRT (**a**) and atypical RA flutter around scar tissue as a function of pacing electrode placement (see Fig. 4). The distance of 0* refers to the electrode located in the slow path (P5 in Fig. 4) The PPI–TCL intervals for the atypical flutter around a scar are lower than in case of AVNRT, mainly because all the pacing points were closer to the atypical flutter circuit than to the CS area. The rise of the PPI–TCL in the slow conduction pathway for the atypical RA flutter is visible. Measuring the PPI–TCL in the proximal (possibly slow-conducting pathway), the distal and at a mid-distance from the CS may help in the rapid recognition of the type of AVNRT. If the PPI–TCL would not decrease with the distance from the slow pathway as in (**b**), it would suggest an atrial tachycardia location not involving the slow conducting pathway—thus not AVNRT. A monotonic change with the distance from CS is typical for AVNRT while a nonmonotonic change indicates another kind of atrial tachycardia

Results of simulation of the atrio-ventricular nodal reentrant tachycardia with Fenton-Karma model are presented in Fig. 5. The AVNRT cycle length obtained in the simulation was 231 ms. The atypical RA flutter around scar cycle length was 212 ms. For the AVNRT, entrainment pacing of 220 ms was chosen, for the atypical RA flutter −200 ms. Five entrainment electrode placements were chosen for the simulation of AVNRT and atypical RA flutter around scar tissue. Four electrode pacing sites were chosen above the coronary sinus near the fast path of conduction and one (P5) in the slow conduction pathway. For each entrainment pacing, the time interval between the last pacing and the next activation at an entrainment electrode was measured and corrected for the tachycardia cycle length (TCL). Calculated PPI–TCL intervals from the simulation of entrainment pacing are shown in Fig. 6.

Two differences between PPI–TCL intervals for both simulations are visible in Fig. 6. The PPI–TCL intervals for the atypical flutter around a scar are lower than in the case of AVNRT—this is mainly because all the pacing points were closer to the atypical flutter circuit than to the CS area. The second difference—the increase of the PPI–TCL in the slow conduction pathway—seems more interesting. Measuring the PPI–TCL response in the proximal and distal CS for a rapid distinction of the left from the right atrial tachycardias was proposed by Miyazaki et al. [23]. The study showed a high sensitivity of distinguishing between common flutter from different lateral RA circuits. The results presented here shows that also measuring the PPI–TCL in the proximal (possibly the low-conducting pathway), the distal and at a mid-distance from CS could help in the rapid recognition of the type of AVNRT. If the PPI–TCL would not decrease with the distance from the slow pathway like in Fig. 6b, it would suggest an atrial tachycardia location not involving the slow conducting pathway—thus not an AVNRT. A monotonic change with the distance from CS is typical for AVNRT while a nonmonotonic change indicates another kind of atrial tachycardia.

### AV orthodromic reciprocating tachycardia

Atrio-ventricular orthodromic reciprocating tachycardia (ORT) is a repetitive re-entrant tachycardia similar to AVNRT. A normal ventricular excitation via the atrio-ventricular node is followed by a delayed reverse excitation of the atria via a pathological, retrograde pathway. The effect may be interpreted as due to the existence of an ectopic source in the atria triggered by a ventricular activation, after a fixed delay. From the point of view of simulation, the phenomenon may be described as a ventricular triggered ectopic source situated in the right atrium. We investigated the alternans effect in the AV conduction time. An experimental study of this effect was presented by Christini et al. [6]. This in vitro study was focused on the functional properties of the tissue of the atrioventricular node, in which an alternans of the conduction during ORT may appear. The study by Christini et al. suggested, that an alternans in the conduction from the atria and to the ventricles can occur even in a single pathway. A similar effect was also studied in [33].Here we test this hypothesis in our model. Only the results from the two dimensional geometry with the FitzHugh–Nagumo model of tissue is shown, as the studies in the cylindrical geometry and Fenton–Karma model yield quantitatively similar results.

#### Simulation protocol

The tachycardia was simulated via a protocol called fixed-delay stimulation. The right atrium was stimulated (at the time denoted *A*) at a fixed time interval *t* = *VA* after the detection of ventricular activation (which occurred at time *V*). Using a fixed delay is consistent with the nearly constant retrograde conduction time that accompanies ORT arrhythmia. AVN recovery time was equal to the VA delay time plus a constant time for the conduction from the atrium retrograde activation site to the AV node. The *t* = *VA* delay time was changed from 40 to 10 ms to manipulate the recovery time and, as a consequence, affect the AVN conduction dynamics. However, at the beginning of each run, VA was set to the initial value of 70 ms and held so until a steady stationary conduction time was established—to provide the same initial conditions for every retrograde stimulation time VA studied. Consecutive interspike intervals were recorded at the output of the AV node. The sinoatrial node, although present in the study, did not affect the behaviour of the system, as it was suppressed by a relatively fast retrograde activation of the right atrium.

**Fig. 7:**
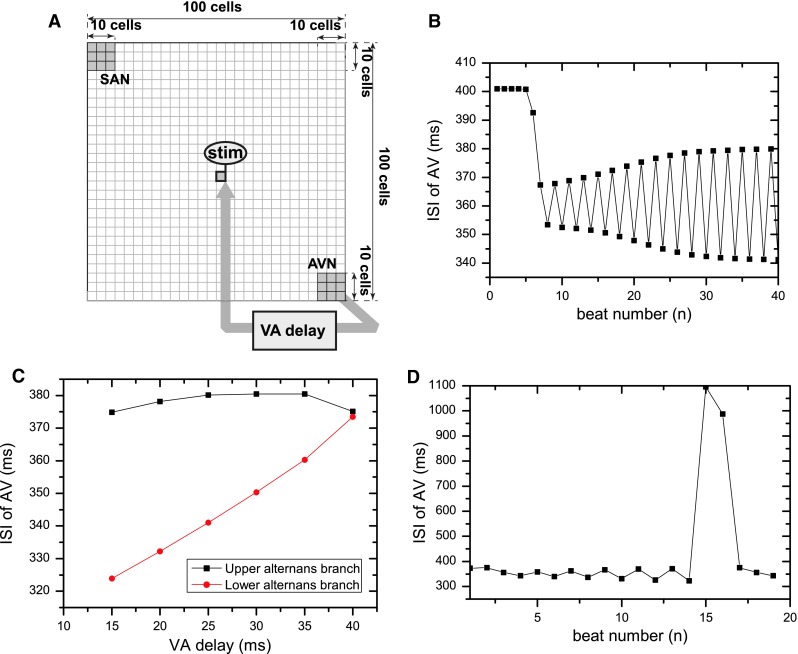
**a** A schematic showing the model of the right atrium. During normal conduction action potential travels from the sinoatrial node (SAN,* upper-left corner* of the picture, through the right atrium (*RA*) to the atrio-ventricular node (*AVN*,* bottom-right corner* of the picture). In the absence of an abnormal retrograde pathway, orthodromic reciprocating tachycardia can be simulated by fixed-delay stimulation of the right atrium after detection of ventricular activation with a delay t = VA. **b** When the VA delay was changed from 70  to 30 ms, the AV intervals rapidly changed and then bifurcated into a beat-to-beat alternans. **c** The alternans in a function of the VA delay. After changing the VA delay interval to 40 ms and less, the AV intervals gradually bifurcated into the alternans. It can be seen that for smaller delay intervals, a larger alternans is obtained. **d** A sequence of ISI obtained for VA decrease to 10 ms. A pattern with a widening alternans leading to a conduction block (in which the AV intervals extend to the top of the graph and AV node produces an action potential of 1,100 ms), and resetting of the pattern by potential wave from SAN (interval no. 17) can be seen. Similar behaviours to those shown in this figure were recorded in some patients in vivo by Christini et al. [6]

The protocol for the simulation was designed to reflect the properties of the in vitro protocol used in [6]. A schematic of the simulation is shown in Fig. 7a.

#### Simulation results

After changing the VA delay interval to 40 ms and less, the AV intervals gradually bifurcated into the alternans (the interspike intervals (ISI) recorded at the AV node can be seen in Fig. 7b, c). After a transient time equal to 20–30 intervals for each VA delay time, a steady alternans was established in every case (the alternans as a function of the VA delay is depicted in Fig. 7c). Due to the bifurcation of the AV conduction time occurring below* VA* = 40 ms two branches can be seen (marked upper and lower branch in Fig. 7c). As the VA delay was shortened, the amplitude of the alternans of the conduction time increased up to 375–324 ms at * VA* = 15 ms. For even shorter VA delay times, the behaviour shown in Fig. 7d occurred. A repetitive pattern consisting of a transient increase in the alternans amplitude, a conduction block during which the AV node produces an action potential (an escape beat), and a resetting of the pattern by a potential wave arriving from the SAN occurred.

Qualitatively similar behaviours to those shown in Fig. 7 were recorded in some patients in vivo by Christini et al. [6]. For one of the patients with the * VA* = 30 ms an alternans with the minimum AV interval of 280 ms and the maximum of 340 ms was recorded. This is consistent with our study showing the alternans below * VA* = 40 ms. The resetting pattern shown in Fig. 7d was also recorded in some patients in vivo by Christini et al.

The conclusion drawn there, that an alternans in the conduction from the atria and to the ventricles can occur even in a single pathway, is supported by the results presented here. Moreover, in the experimental study presented by Christini et al., the application of an autonomic blockade did not prevent the alternans from occurring but even evoked it in three patients who did not have a preblockade alternans.

Such a single-pathway alternans may appear because of the specific restitution properties of the tissue. To check these properties, we simulated the APD and CV restitution curves [2] for the AVN nodal tissue. The APD restitution curve is the Action Potential Duration (APD) as a function of the external stimulation cycle length (CL) [20].

**Fig. 8:**
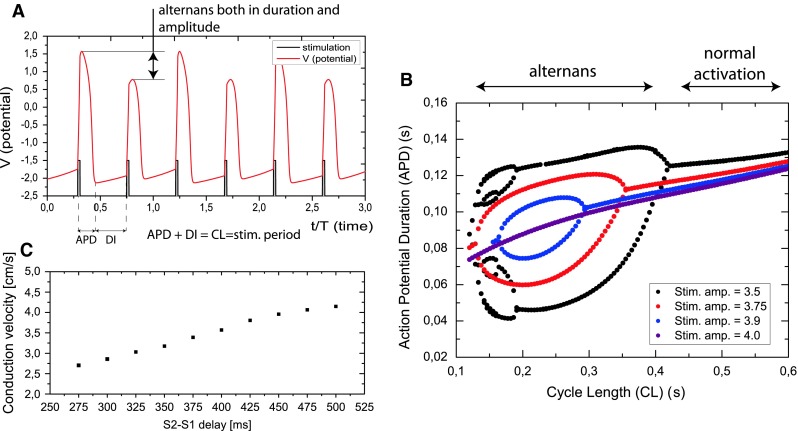
Restitution properties of nodal tissue model. We apply the dynamic restitution protocol [20] to an isolated single cell to obtain the APD vs cycle length (*CL*) restitution curve , and the S1−S2 restitution protocol to obtain the CV restitution curve.** a** Action potential versus time for the fixed pacing cycle length. One cell of the model was stimulated externally at a fixed cycle length (*CL*) until a steady state was reached. All action potential duration (*APD*) values obtained for the corresponding cycle length (*CL*) were recorded with a time window length kept fixed in order not to exclude multiple bifurcations.** b** APD versus CL for different stimulation amplitudes. All the recorded APD and CL pairs were plotted on this graph. For longer stimulation periods, a flat, non-alternating APD response can be seen. For stimulation periods shorter than 400 ms the APDs begins to alternate—a bifurcation occurs. The position of the bifurcation point depends on the strength of stimulation.** c** The conduction velocity (*CV*) versus the S2–S1 time. The action potential duration versus cycle length is plotted for different stimulation strengths. The CV restitution curve for the nodal tissue model was measured in a tissue strip of 8 cm × 0.32 cm using the S1–S2 restitution protocol: after the intrinsic activation of the AVN, an external stimulus was delivered to one end of the tissue strip at a time S1 and a second stimulus at the time S2. The time of propagation through the tissue strip was measured and divided by the tissue strip length to obtain conduction velocity

The abundance of different protocols for the estimation of the restitution curve is often the source of misinterpretations of the results. Here, we apply the dynamic restitution protocol to an isolated, single cell to obtain the APD vs cycle length (CL) restitution curve [20]. Each pair of APD and CL values is obtained by pacing at a fixed cycle length until a steady state is reached (an example of such a state is presented in Fig. 8a on a time vs potential graph). During alternans, all APD and CL pairs are recorded in a fixed time window (in order to not exclude multiple bifurcations and period-n oscillations). In Fig. 8b, the action potential duration versus cycle Length is plotted for different stimulation strengths. The CV restitution curve for the nodal tissue model was measured in a tissue strip of 8 cm × 0.32 cm using the S1–S2 restitution protocol: after the intrinsic activation of AVN, an external stimulus was delivered to one end of the tissue strip at a time S1, and a second stimulus at time S2. The time of propagation through the tissue strip was measured and divided by the tissue strip length to obtain conduction velocity. The conduction velocity (CV) versus S2–S1 time is plotted in Fig. 8c.

In the Fig. 7c, it can be seen that the conduction alternans occurs for the interspike intervals smaller then 380 ms. In Fig. 8b, the APD alternans occurs when the stimulation cycle length is below 400 ms, depending on the amplitude of stimulation. It is difficult to compare the external stimulation in the APD/CL restitution curve simulation and the excitation of the AVN by the surrounding atrial tissue in terms of the total current delivered. However, qualitatively, the fast reverse excitation of the atria acts on the nodal tissue similarly to rapid pacing. It seems that the fast excitation of the AVN in the case of the orthodromic tachycardia leads to an alternans in the duration of the APD of the AVN cells, which is correlated with an alternans of the conduction velocity. This supports the hypothesis that the restitution properties of the AV node, and not the autonomic nervous system activity, is the main reason for the conduction alternans during AV orthodromic reciprocating tachycardia. And that only a single conducting pathway is necessary for this alternans to occur.

### Atrial parasystole

Atrial parasystole is a supraventricular arrhythmia caused by the presence of a secondary, ectopic pacemaker in the atrium, which operates independently of the SAN and is not suppressed by its activity. Usually, the period of the parasystole is smaller than that of the sinus rhythm. Below we discuss how, depending on the location of the parasystolic pacemaker, shortened or lengthened RR intervals may occur in the tachogram of a parasystolic rhythm. The possibility of locating the parasystolic source will be studied in two geometrical settings of the RA—the two dimensional tissue strip with the FitzHugh–Nagumo model of the tissue and the cylindrical idealized geometry of the RA with the Fenton–Karma model of the tissue. Two cases of atrial parasystole were described in [15]. It was suggested there that the atrial parasystolic pacemaker may sometimes lie within the atrial preferential conducting pathway. We would like to check this possibility in simulation.

#### Simulation protocol

**Fig. 9:**
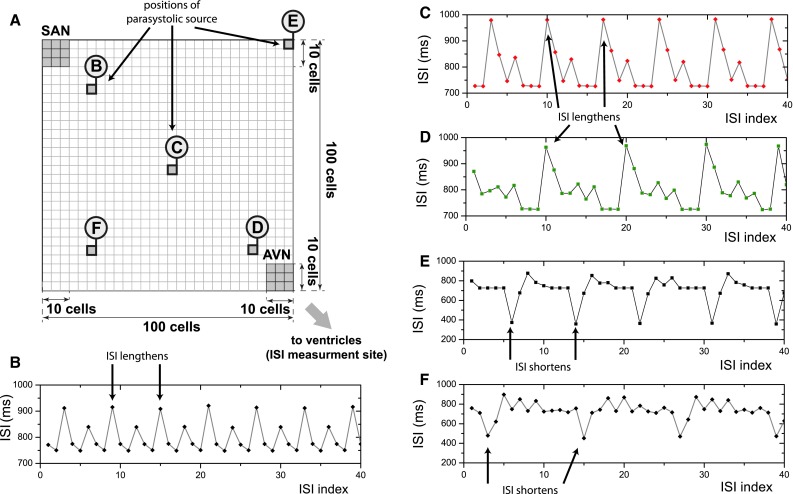
**a** Schematic drawing showing different locations for the parasystolic ectopic source for the cases** b**–**f**. **b**−**d** For all the locations on the line joining the nodes, a lengthening of the ISI measured at the exit of AVN occurs. **e**–**f** For all the locations away from the line joining the nodes, a shortening of the ISI measured at the exit of AVN occurs

**Fig. 10:**
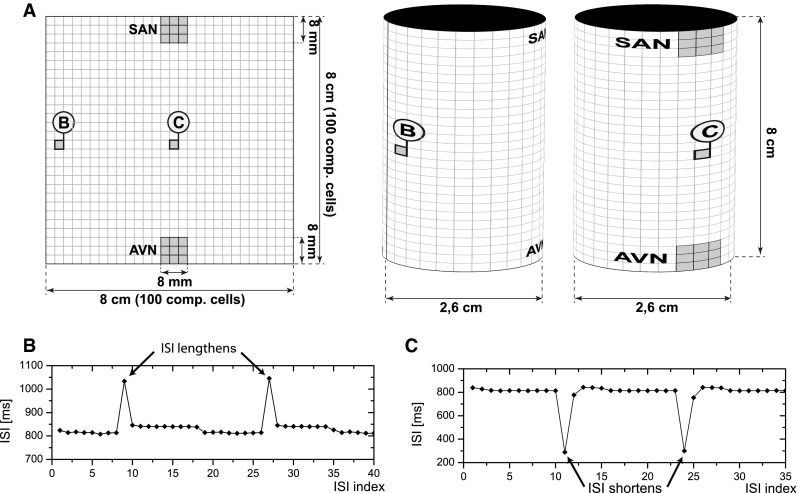
Schematic drawing showing different locations for the parasystolic ectopic source for the cases** b** and** c**. **b** For all the locations on the line joining the nodes, a lengthening of the ISI measured at the exit of AVN occurs. **c** For all the locations away from the line joining the nodes, a shortening of the ISI measured at the exit of AVN occurs

Five different locations were chosen within the atrium for the parasystolic ectopic source, as in Figs. 9a and 10a. For the study, in Fig. 9 the period of SAN activity was set to $$T = 750$$ ms and the period of the parasystolic activity to $$T = 800 $$ ms. For the study in Fig. 10 the period of SAN activity was set to $$T = 813$$ ms and the period of the parasystolic activity to $$T = 840 $$ ms. Both remained constant for all cases studied below. Different properties of the ISI interval time series measured at the exit of the AVN were obtained, depending on the whether the parasystolic source was located on the fast conduction pathway joining the nodes or at a location distant from this line. It should be stressed that the main reason for the differences in the properties of the ISI time series obtained in our study will be the difference between the refractory period in the AVN tissue and that of the normal, conducting atrial tissue irrespective of the type of model and geometry used.

#### Simulation results

**Fig. 11:**
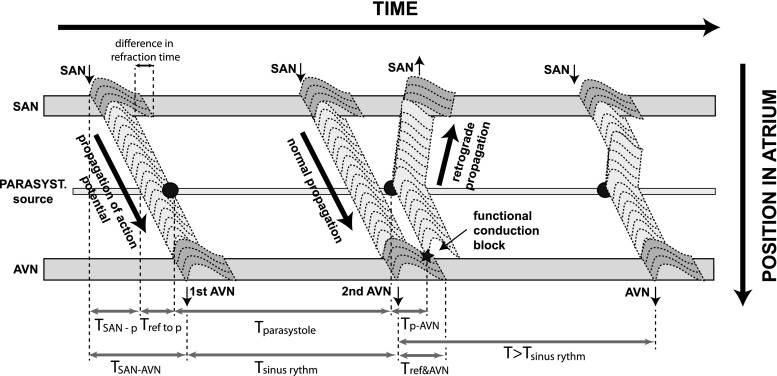
A Lewis diagram for the parasystolic activity. A parasystolic ectopic source lies in the middle of the conduction line between sinoatrial and atrio-ventricular node (marked by a* black disk*. The vertical coordinate represents the position in the atrium on the conduction line and the time is horizontal. At first, a normal conduction like during sinus rhythm occurs. During the time *T*_*SAN*−*AVN*_, the action potential travels from the sinoatrial node to the atrio-ventricular node. The refractory time of the tissue is represented by the* gray sketch* of the action potential shape; it is longer at the nodal tissue. The ectopic activity during this first passage of the SAN action potential wave is suppressed (not reset) by the refraction of the surrounding tissue. Right after the second propagation of the action potential through the atrium, the parasystolic source produces an action potential wave—*T*_*sinus* *rhythm*_ < *T*_*parasystole*_ . However, the activation wave coming from parasystolic source fails to enter the AVN node (denoted ‘functional conduction block’). Due to that, the next activation of the ventricles will be delayed, producing a longer interspike interval, as in Fig. 9. Note that the velocity of the retrograde conduction and that of the conduction in the forward direction are the same, and that the difference visible in the figure is due to the perspective

Whenever the ectopic source is located on the preferential conducting pathway, a lengthening of the ISI measured at the exit of the atrio-ventricular node occurrs (Figs. 9b–d, 10b). Moreover, the transient behaviour between the instances of elongated ISIs were different and depended on the location of the ectopic source. While for the model of a single cell there is no intrinsic memory longer than one period of activity, it seems that the coupling of the cells in the model of the atrium introduces a memory effect resulting in a pattern lasting several ISI. For all the locations of the ectopic source away from the line joining the nodes, a shortening of the ISI measured at the exit of the AVN occurs (Figs. 9 e, f, 10c). The observed difference between the shortening and the lengthening of ISI for different locations of the parasystolic source may be understood by means of Fig. 11 which depicts the propagation of action potentials in the atrium for location C of Fig. 9: the parasystolic ectopic source then lies in the middle of the conduction line between the sinoatrial and the atrio-ventricular node and is marked by a black disk filled circle. The propagation of the action potential is presented on a Lewis diagram, where the vertical coordinate is the position in the atrium along the conduction line, and the horizontal coordinate is the time. At first, a normal conduction as in sinus rhythm occurs. During the time *T*_*SAN*−*AVN*_, the action potential travels from the sinoatrial node to the atrio-ventricular node. The refractory time of the tissue is represented in Fig. 11 by the length of the action potential shape and it is longer inside the nodal tissue. The ectopic activity is suppressed (not reset) by the refraction of the surrounding tissue. Right after the second propagation of the action potential through the atrium, the parasystolic source produces an action potential wave and *T*_*sinus rhythm*_ < *T*_*parasystole*_. However, the activation wave coming from the parasystolic source fails to enter the AVN node (marked ’functional conduction block’ in Fig. 11). Due to that, the next activation of the ventricles will be delayed, producing a longer interspike interval, as in Fig. 9. The following condition for the lengthening of the ISI interval for the propagation times between the elements of the model holds in the case presented in Fig. 11:

5 $$ T_{SAN-AVN} + T_{sinus \, rhythm} + T_{ref\&AVN} > T_{SAN-p} + T_{ref \, top} + T_{parasys.}+ T_{p-AVN} $$

where *T*_*SAN*-*AVN*_ is the time of propagation from SAN to AVN, *T*_*sinus rhythm*_ is the sinus rhythm period, $T_{ref\&AVN}$ is the refraction period at AVN, *T*_*SAN*−*p*_ is the time of propagation from SAN to the parasystolic source, *T*_*reftop*_ is the time at the location of the parasystole between the external excitation of the tissue and parasystolic activity, *T*_*parasystole*_ is the period of parasystolic activity, *T*_*p*−*AVN*_ is the time of propagation from the parasystolic source to AVN.

The condition (5) may not hold only if the parasystolic source is away from the main conduction line between SAN and AVN. Then, occasionally, a shorter ISI will occur, as in Fig. 9 e, f. The minimal distance between the parasystolic source and the main conduction line for which a shorter ISI will occur depends on the period of the parasystolic activity. From the condition (5) we can tell, whether the parasystolic source lies on the pathway directly connecting the SAN with the AV node (the normal conduction pathway) or whether it is located away from it. If the period of parasystolic activity $T_{parasystole} > T_{sinus rhythm}+ T_{ref\&AVN},$ the ISI will not be lengthened.

Note that the short ISI visible in Fig. 9e, f in a real tachogram would be readily recognized as the result of arrhythmia due to their length. On the other hand, the the elongated ISI in Fig. 9b, c would not. The latter are then a kind of concealed conduction patterns. We expect that very similar patterns may be obtained in patients with very low heart rate variability and a parasystole. The effect of breathing on the variability of the ISI in the right atrium containing a parasystole will be the subject of a separate paper.

## Conclusions

In order to improve our understanding of the very complex phenomena that occur in the conduction system of the heart, we have, as a first step, to find techniques which allow to study the underlying physics occurring in the model systems, in a feasible, robust way. We found that Liénard equations may be used to describe the pacemaker tissue of the electrical conduction system in a uniform and flexible way. What is important, future analysis using these equations may be performed on a scale of thousands of heartbeats—a scale at which the study of heart rate variability becomes feasible. The novel inclusion of the nodal tissue into the same model together with the atrial muscle tissue enables to study the relations between an ectopic state of a part of the tissue and the properties of the natural pacemakers of the heart. Models of the sinoatrial and atrio-ventricular nodes reconstruct physiologically important properties such as the alternans in case of a ventricular triggered atrial pacing, or nonlinear behaviour during the AVNRT.

In this study, we found that, to obtain the type II AVNRT (the "fast-slow" mode) in our model, it was enough to disrupt the conduction in the slow pathway by diminishing the refractory period for a finite time. The length of the time this pathway remained disrupted was not important. The phase at which it regained its normal state was, however, crucial. We were able to find such a combination of the refraction periods and diffusion coefficients for which the reentry continued indefinitely. We propose that measuring the PPI–TCL within the proximal (possibly—the slow-conducting pathway), the distal and at a mid-distance from the CS could help in a rapid recognition of AVNRT as different from other atrial tachycardias.

We modeled the AV orthodromic reciprocating tachycardia and we obtained an alternans of conduction time throughout the atrio-ventricular node, similarly to earlier experimental studies by Christini et al. [6]. The conclusion that the alternans in the conduction between the atria and the ventricles can occur within one pathway ([6]) is supported by our study. Moreover, the results discussed in the study show that complex functional properties of the AVN can be simulated in a simple model of the right atrium. These properties include a nonlinear response to the external excitement (by an action potential from the tissue or external stimulation by a catheter) and memory or transient effects obtained in this study during the alternans of the conduction time. Certain nonlinear functional properties were also obtained in the phase response analysis and restitution analysis of the atrio-ventricular node model. These have been submitted elsewhere.

In the atrial parasystole simulation, we found a mathematical condition which allows for a rough estimation of the location of the parasystolic source within the atrium, both for the simplified planar and cylindrical geometry of the atrium. We can tell, whether the parasystolic source lies on the pathway directly connecting the SAN with the AVN node (the normal conduction pathway) or whether it is located away from it. Some of the results in the simulation of the atrial parasystole in a real tachogram would be readily recognized as the result of the arrhythmia due to their length. On the other hand, some patterns obtained by us should be considered concealed conduction patterns. We expect that very similar patterns may be obtained in patients with a very low heart rate variability and a parasystole.

The models used in the simulations presented here are very simplified. Both the 2D planar geometry as well as the cylindrical geometry used for the atrium as well as the FitzHugh–Nagumo and Fenton–Karma equations are much simpler than the true geometry and the physiology of the atrium. However, in spite of these simplifications, our models were able to reproduce several phenomena obtained in the electrophysiology lab. Moreover, the results obtained from the FitzHugh–Nagumo planar model and the cylindrical geometry of the Fenton–Karma model are, to a large extent, equivalent. This indicates that such simple models may be useful for testing certain hypotheses on the pathophysiology of the atrium. We present and verify assumptions of the nodal electrical activity model integrated with atrial muscle tissue. Such setting of the modelling framework will allow in the future to study phenomena on a time-scale of thousands of heartbeats. Simulations of long time series should allow to test and develop new HRV measures.

## Abbreviations

AF: Atrial fibrillation
AFL: Atrial flutter
APD: Action potential duration
AVN: Atrio-ventricular node
BCL: Basic cycle length
CS: Coronary sinus
CV: Conduction velocity
DI: Diastolic interval
EP: Electrophysiology
ERP: Effective refractory period
ISI: Interspike interval—interval between two consecutive cell activations
PPI: Post-pacing interval
RA: Right atrium
SAN: Sinoatrial node
SVT: Supraventricular tachycardia
VA: Delay ventriculo-atrial delay
TCL: Tachycardia cycle length

## References

[1] Almendral J, Stamato N, Rosenthal M, Marchlinski F, Miller J, Josephson M (1986). Resetting response patterns during sustained ventricular tachycardia: relationship to the excitable gap. Circulation.

[2] Banville I, Gray RA (2002). Effect of action potential duration and conduction velocity restitution and their spatial dispersion on alternans and the stability of arrhythmias. J Cardiovasc Electrophysiol.

[3] Bartocci E, Cherry E, Glimm J, Grosu R, Smolka SA, Fenton FH. Toward real-time simulation of cardiac dynamics. In: CMSB 2011: Proceedings of the 9th ACM International Conference on Computational Methods in Systems Biology. Paris, France: ACM; 2011. p. 103–110.

[4] Carusi A, Burrage K, Rodríguez B. Bridging experiments, models and simulations: an integrative approach to validation in computational cardiac electrophysiology. Am J Physiol Heart Circ Physiol. 2012;303(2):H144–55. http://www.ncbi.nlm.nih.gov/pubmed/22582088. 10.1152/ajpheart.01151.2011 22582088

[5] Cherry EM, Fenton FH (2010). Realistic cardiac electrophysiology modelling: are we just a heartbeat away. The Journal of Physiology.

[6] Christini DJ, Stein KM, Markowitz SM, Mittal S, Slotwiner DJ, Iwai S, Lerman BB. Complex AV nodal dynamics during ventricular-triggered atrial pacing in humans. Am J Physiol Heart Circ Physiol. 2001;281(2):H865–72. http://www.ncbi.nlm.nih.gov/pubmed/11454592. 10.1152/ajpheart.2001.281.2.H865 11454592

[7] Comtois P, Nattel S. Impact of tissue geometry on simulated cholinergic atrial fibrillation: a modeling study. Chaos: an Interdisciplinary. J Nonlinear Sci. 2011;21(1):013108. doi:10.1063/1.3544470. http://link.aip.org/link/?CHA/21/013108/1. 10.1063/1.3544470 21456822

[8] Cosio FG, Martin-Penato A, Pastor A, Nunez A, Goicolea A (2003). Atypical flutter: a review. Pacing Clin Electrophysiol.

[9] Courtemanche M, Ramirez RJ, Nattel S (1998). Ionic mechanisms underlying human atrial action potential properties: insights from a mathematical model. American Journal of Physiology - Heart and Circulatory Physiology.

[10] Derejko P, Szumowski ŁJ, Sanders P, Dimitri H, Kuklik P, Przybylski A, Urbanek P, Szufladowicz E, Bodalski R, Sacher F, Haïssaguerre M, Walczak F. Clinical validation and comparison of alternative methods for evaluation of entrainment mapping. J Cardiovasc Electrophysiol. 2009;20(7):741–8. doi:10.1111/j.1540-8167.2008.01425.x. 10.1111/j.1540-8167.2008.01425.x 19207782

[11] Dössel O, Krueger MW, Weber FM, Wilhelms M, Seemann G. Computational modeling of the human atrial anatomy and electrophysiology. Med Biol Eng Comput. 2012;50(8):773–99. http://www.ncbi.nlm.nih.gov/pubmed/22718317. 10.1007/s11517-012-0924-6 22718317

[12] Fenton F, Karma A. Vortex dynamics in three-dimensional continuous myocardium with fiber rotation: filament instability and fibrillation. Chaos (Woodbury, NY). 1998;8(4):20–47. http://www.ncbi.nlm.nih.gov/pubmed/12779795. 10.1063/1.166311 12779708

[13] FitzHugh R (1961). Impulses and Physiological States in Theoretical Models of Nerve Membrane. Biophysical journal.

[14] Forest L, Glade N, Demongeot J (2007). Liénard systems and potential-Hamiltonian decomposition: applications in biology. Comptes rendus biologies.

[15] Friedberg HD, Schamroth L (1970). Atrial parasystole. British heart journal.

[16] Garcia-Cosio F, Pastor Fuentes A, Nunez Angulo A (2012). Clinical Approach to Atrial Tachycardia and Atrial Flutter From an Understanding of the Mechanisms. Electrophysiology Based on Anatomy. Revista Espanola De Cardiologia.

[17] Grandi E, Pandit SV, Voigt N, Workman AJ, Dobrev D, Jalife J, Bers DM (2011). Human atrial action potential and Ca2+ model: sinus rhythm and chronic atrial fibrillation. Circulation research.

[18] Heidbüchel H. How to ablate typical ‘slow/fast’ AV nodal reentry tachycardia. Europace. 2000;2(1):15–9. doi:10.1053/eupc.1999.0070. http://www.ncbi.nlm.nih.gov/pubmed/11225592. 10.1053/eupc.1999.0070 11225592

[19] Inada S, Hancox JC, Zhang H, Boyett MR (2009). One-dimensional mathematical model of the atrioventricular node including atrio-nodal, nodal, and nodal-his cells. Biophysical journal.

[20] Koller M, Riccio M, RF GJ (1998). Dynamic restitution of action potential duration during electrical alternans and ventricular fibrillation. Am J Physiol.

[21] Lienard A. Etude des oscillations entretenues. Revue generale de l’electricite. 1928;23:901–12;946–54.

[22] Roger VL, Go AS. Heart disease and stroke statistics? 2012 update. Circulation. 2012;125(1):e2–220. doi:10.1161/CIR.0b013e31823ac046. http://circ.ahajournals.org/content/125/1/e2.short, http://circ.ahajournals.org/content/125/1/e2.full.pdf+html. 10.1161/CIR.0b013e31823ac046 PMC4440543 22179539

[23] Miyazaki H, Stevenson WG, Stephenson K, Soejima K, Epstein LM. Entrainment mapping for rapid distinction of left and right atrial tachycardias. Heart Rhythm. 2006;3(5):516–23. doi:10.1016/j.hrthm.2006.01.014. http://www.sciencedirect.com/science/article/pii/S1547527106000701. 10.1016/j.hrthm.2006.01.014 16648054

[24] Munoz MA, Kaur J, Vigmond EJ (2011). Onset of atrial arrhythmias elicited by autonomic modulation of rabbit sinoatrial node activity: a modeling study. American Journal of Physiology - Heart and Circulatory Physiology.

[25] Oliveira M, da Silva MN, Geraldes V, Xavier R, Laranjo S, Silva V, Postolache G, Ferreira R, Rocha I (2011). Acute vagal modulation of electrophysiology of the atrial and pulmonary veins increases vulnerability to atrial fibrillation. Exp Physiol.

[26] Postnov D, Han SK, Kook H (1999). Synchronization of diffusively coupled oscillators near the homoclinic bifurcation. Phys Rev E.

[27] Roberts BN, Yang PC, Behrens SB, Moreno JD, Clancy CE. Computational approaches to understand cardiac electrophysiology and arrhythmias. Am J Physiol Heart Circ Physiol. 2012;303(7):H766–83. doi:10.1152/ajpheart.01081.2011. 10.1152/ajpheart.01081.2011 PMC3774200 22886409

[28] Rudski LG, Lai WW, Afilalo J, Hua L, Handschumacher MD, Chandrasekaran K, Solomon SD, Louie EK, Schiller NB. Guidelines for the echocardiographic assessment of the right heart in adults: a report from the American Society of Echocardiography endorsed by the European Association of Echocardiography, a registered branch of the European Society of Cardiology, and the Canadian Society of Echocardiography. J Am Soc Echocardiogr. 2010;23(7):685–713; quiz 786–8. http://www.ncbi.nlm.nih.gov/pubmed/20620859. 10.1016/j.echo.2010.05.010 20620859

[29] Sánchez C, Corrias A, Bueno-Orovio A, Davies M, Swinton J, Jacobson I, Laguna P, Pueyo E, Rodríguez B. The Na+/K+ pump is an important modulator of refractoriness and rotor dynamics in human atrial tissue. Am J Physiol Heart Circ Physiol. 2012;302(5):H1146–59. http://www.ncbi.nlm.nih.gov/pubmed/22198174. 10.1152/ajpheart.00668.2011 PMC3311461 22198174

[30] Smith N, de Vecchi A, McCormick M, Nordsletten D, Camara O, Frangi AF, Delingette H, Sermesant M, Relan J, Ayache N, Krueger MW, Schulze WHW, Hose R, Valverde I, Beerbaum P, Staicu C, Siebes M, Spaan J, Hunter P, Weese J, Lehmann H, Chapelle D, Rezavi R (2011). euHeart: personalized and integrated cardiac care using patient-specific cardiovascular modelling. Interface focus.

[31] Stavrakis S, Scherlag BJ, Po SS (2012). Autonomic modulation. Circulation: Arrhythmia and Electrophysiology.

[32] Stevenson W, Khan H, Sager P, Saxon L, Middlekauff H, Natterson P, Wiener I (1993). Identification of reentry circuit sites during catheter mapping and radiofrequency ablation of ventricular tachycardia late after myocardial infarction. Circulation.

[33] Sun J, Amellal F, Glass L, Billette J (1995). Alternans and period-doubling bifurcations in atrioventricular nodal conduction. Journal of theoretical biology.

[34] Winner M, Augostini R, Houmsse M. Differentiation of narrow complex tachycardia. Ibnosina J Med. 2011;3(1):32–5. http://journals.sfu.ca/ijmbs/index.php/ijmbs/article/viewArticle/166.

[35] Żebrowski JJ, Grudziński K, Buchner T, Kuklik P, Gac J, Gielerak G, Sanders P, Baranowski R. Nonlinear oscillator model reproducing various phenomena in the dynamics of the conduction system of the heart. Chaos(Woodbury, NY). 2007;17(1):015121. http://www.ncbi.nlm.nih.gov/pubmed/17411278. 10.1063/1.2405128 17411278

[36] Żebrowski JJ, Kuklik P, Buchner T, Baranowski R. Concealed conduction effects in the atrium: a unified model of the atrium, sinoatrial, and atrioventricular nodes. IEEE Eng Med Biol Mag. 2009;28(6):24–9. doi:10.1109/MEMB.2009.934628. 10.1109/MEMB.2009.934628 19914884

[37] Zemlin C, Mitrea B, Pertsov A. Spontaneous onset of atrial fibrillation. Physica D. 2009;238:969–75. http://www.sciencedirect.com/science/article/pii/S016727890800420X. 10.1016/j.physd.2008.12.004 PMC2768313 20160895

